# Computational Modeling of Doped 2D Anode Materials for Lithium-Ion Batteries

**DOI:** 10.3390/ma16020704

**Published:** 2023-01-11

**Authors:** Alexander Galashev

**Affiliations:** 1 Institute of High-Temperature Electrochemistry, Ural Branch, Russian Academy of Sciences, Akademicheskaya Str. 20, Yekaterinburg 620066, Russia; galashev@ihte.uran.ru; 2 Institute of Chemical Engineering, Ural Federal University Named after the First President of Russia B.N. Yeltsin, Mira Str., 19, Yekaterinburg 620002, Russia

**Keywords:** copper, first-principle calculations, graphite, lithium ion battery, molecular dynamics, nickel, nitrogen, silicene, spectrum of electronic states, transmutation doping

## Abstract

Development of high-performance lithium-ion batteries (LIBs) is boosted by the needs of the modern automotive industry and the wide expansion of all kinds of electronic devices. First of all, improvements should be associated with an increase in the specific capacity and charging rate as well as the cyclic stability of electrode materials. The complexity of experimental anode material selection is now the main limiting factor in improving LIB performance. Computer selection of anode materials based on first-principles and classical molecular dynamics modeling can be considered as the main paths to success. However, even combined anodes cannot always provide high LIB characteristics and it is necessary to resort to their alloying. Transmutation neutron doping (NTD) is the most appropriate way to improve the properties of thin film silicon anodes. In this review, the effectiveness of the NTD procedure for silicene/graphite (nickel) anodes is shown. With moderate P doping (up to 6%), the increase in the capacity of a silicene channel on a Ni substrate can be 15–20%, while maintaining the safety margin of silicene during cycling. This review can serve as a starting point for meaningful selection and optimization of the performance of anode materials.

## 1. Introduction

Lithium-ion batteries (LIBs) have many applications, ranging from portable electronic devices to electric vehicles [1]. At present, graphite is still the main anode material for lithium-ion batteries (LIBs). When used in this capacity, graphite has certain advantages, such as low cost, absolute abundance, non-toxicity, and structural stability [2,3,4]. However, the graphite electrode has a low capacitance (~370 mAh/g), and it may intereact with the electrolyte at low operating voltages, resulting in lithium deposition [5,6,7]. In this case, the performance of the battery is reduced, and the safety hazard is increased. The search for alternative anode materials is necessary to improve the electrochemical properties of LIBs.

Increased capacitance, high energy density and improved cycle characteristics for LIBs can be obtained through the use of new anode materials [8]. Silicon occupies a leading position among them because it has the highest specific capacity of 4200 mAh/g [9] among known materials. Other advantages of Si as an anode material are the low operating potential (0.5 V vs. Li/Li^+^), natural abundance, and its environmental benignity.

A large change in the volume (~300%) of the anode during cycling, which ultimately leads to its destruction, hinders the practical use of anodes made of massive silicon materials. The service life of a silicon anode can be increased by replacing bulk silicon with thin films of silicon. Films with thicknesses ranging within a nanometer are especially attractive [10]. The use of thin films significantly improves the characteristics of the electrodes, increasing their resistance to cycling and significantly reducing the volume expansion during lithiation.

Graphene and silicene have sufficient strength and hence may be reasonably used to manufacture flexible electrodes [11]. The high tendency of silicene to oxidize in air is a certain obstacle. However, this problem can be solved, for example, by using Al_2_O_3_ protective layers [12]. Al_2_O_3_-based encapsulation can be carried out when silicene is present on any substrate. This achieves an atomically clear and chemically intact Al_2_O_3_/silicene interface.

As is known, silicene and its bulk counterpart do not exist in nature and can be obtained by high vacuum epitaxial deposition on substrates. So far, silicene has been synthesized on several substrates such as Ag(110) [13,14], Ag(111) [15,16,17], Au(110) [18], ZrB_2_-covered Si(111) [19], Ir(111) [20], and MoS_2_ [21]. Thus, silicene is not some kind of virtual material.

As a rule, doping of semiconductors is used to significantly change their electrical and optical properties. As a result of doping, impurity states are created in the band gap of a semiconductor, which affects its physical properties. By adjusting the doping level, one can change the values of electrical and photoluminescent properties [22,23]. In particular, doping can increase the conductivity of a thin crystalline silicon film [24].

Increasing the electrical conductivity of the anode material improves the charging rate of LIBs. Doping of silicene with phosphorus (an element of group V) introduces additional uncompensated electrons into the system and, with heavy doping, should significantly increase its electrical conductivity. In addition, it has been experimentally established that in phosphorus-doped silicon, the Si-P bond strength is higher than the Si-Si bond strength [25]. It was shown in [26] that the strength of graphene increases when it is doped with nitrogen.

Investigations based on DFT calculations showed that upon codoping of silicene with Ag, Au, Cu, Ti, Mn, and N its structural stability increases as the number of N atoms increase [27]. Doping of silicene with trivalent boron does not create a high density of two-dimensional free holes but leads to a change in the electronic properties of the entire system, so that the resulting systems exhibit metallic behavior [28]. Doping of silicene with carbon leads to the formation of polar C-Si covalent bonds, which are stronger than nonpolar Si-Si covalent bonds [29]. This causes the appearance of a greater elastic rigidity of the two-dimensional anode material, regardless of the lithium concentration in the interstices. DFT calculations of three-layer aluminum-functionalized silicene show that such a material is promising for the design of LIB anodes, giving a theoretical capacity of 645 mAh g^−1^ [30].

With the help of nuclear transmutation doping (NTD), in one session of irradiation with thermal neutrons, some of the silicon and carbon atoms are converted into phosphorus and nitrogen atoms, respectively. It is also possible to convert Ni atoms into Cu atoms. In our opinion, it is the NTD procedure that is the most effective way to strengthen silicene and to give it a higher electronic conductivity. The proposals outlined in [27,28,29,30] require alternative approaches to doping.

Silicene has been the subject of a large number of papers, including review papers (see, for example, [31,32,33,34]). The possibility of using silicene as an anode material for LIBs was studied by the molecular dynamics method [35,36,37,38,39,40,41,42,43,44,45] and using quantum mechanical calculations based on the density functional theory (DFT) approximation [46,47,48,49,50,51,52,53,54]. However, a thorough analysis of calculations on the use of silicene in energy storage devices has not been conducted.

The purpose of this review is to highlight the works aimed at improving the predicted performance of a silicene anode through transmutation neutron doping. All works related to this area of research are based on quantum mechanical DFT calculations and on modeling by the method of classical molecular dynamics.

## 2. Materials and Methods

There are three important parts of schemas for DFT calculations: pseudopotentials, basis, and k-grid operation. Ultrasoft pseudopotentials were used in the calculations [55]. These pseudopotentials are optimized by minimizing the difference in the results of calculating various electronic configurations obtained in the pseudopotential approximation and taking into account the energy of all electrons. The assignment of the basis set was performed according to the data reported in [55]. A not too dense, and therefore, not very “expensive” mesh, which nevertheless provided acceptable convergence, was used. In the MD ab initio calculations, the Born–Karman periodic boundary conditions were used. The models used are described in more detail in [46,47,48,49,50,51].

DFT studies allow determining properties such as geometries, energies, reaction mechanisms, and spectroscopic properties. DFT results can serve to confirm the conclusions drawn from the analysis of experiments. All DFT calculations used the generalized gradient approximation (GGA) in the PBE form [56], which includes the energy dependence not only on the electron density, but also on its gradient. This makes it possible to describe better the inhomogeneous nature of molecular densities. The energy calculation error when using GGA is typically no more than 0.2 eV [57].

The anode model in classical MD calculations was represented by two-layer doped silicene formed by 600 Si and P atoms and doped graphite (from 3348 C and N atoms) [11,46] and nickel (from 2418 Ni and Cu atoms) [39,45] substrates. Both non-metallic and metallic substrates were formed by 6 layers of atoms. The substrates were facing silicene by close-packed planes. Two-layer silicene formed a flat channel with a gap $h_{g}$ from 0.24 to 0.75 nm, the walls of which were doped with phosphorus. Doping of silicene in the model was carried out as follows: On each sheet of silicene, 9 vacancy defects were uniformly distributed. Among them were mono-, bi-, tri-, and hexavacancies. Each of these vacancy defects was filled with P atoms, which replaced the Si atoms withdrawn during the formation of defects. As a result, silicene sheets containing 3%, 6%, 9%, and 18% P atoms were obtained. The Ni(111) substrate was also doped. To do this, 5% randomly selected Ni atoms were replaced by Cu atoms. In addition, lithium intercalation into the channel was also simulated in the presence of perfect silicene walls in the channel and walls containing the indicated fractions of the vacancy defects considered above.

One lithium ion was launched into the silicene channel every 10 ps. The channel was filled with lithium under the action of a constant electric field (1 × 10^−4^ V/m). The Li^+^ ion that hit the negative electrode (anode) acquired an electron and, after 10 ps, turned into an uncharged Li atom. The number of intercalated lithium atoms reached 140.

The interaction of atoms in silicene and graphene sheets (that form graphite) was described using the Tersoff potential [58]:

(1) $$V_{ij}=f_{C}(r_{ij})\left[A\exp(-\lambda^{\left(1\right)}r_{ij})-Bb_{ij}\exp(-\lambda^{\left(2\right)}r_{ij})\right]$$

$$f_{C}(r_{ij})=\{\begin{array}{c} 1,\\ \frac{1}{2}\\ 0 \end{array}+\frac{1}{2}\cos\left[\pi \left(r_{ij}-R^{\left(1\right)}\right)/(R^{\left(2\right)}-R^{\left(1\right)})\right],\;\begin{array}{c} r_{ij}<R^{\left(1\right)}\\ R^{\left(1\right)}<r_{ij}<R^{\left(2\right)}\\ r_{ij}>R^{\left(2\right)} \end{array},$$

$$b_{ij}={\left(1+\beta^{n}\xi_{ij}^{n_{i}}\right)}^{-1/(2n)},\;\xi_{ij}=\sum_{k\neq i,j}f_{C}(r_{ij})g(\theta_{ijk}),$$

(2) $$g(\theta_{ijk})=1+\frac{c^{2}}{d^{2}}-\frac{c^{2}}{\left[d^{2}+{\left(h-\cos\theta_{ijk}\right)}^{2}\right]}$$

Here, $b_{ij}$ is a multiparticle bond order parameter that describes how the bond formation energy (attractive part $V_{ij}$) is created in a local atomic arrangement due to the presence of other neighboring atoms, *ξ* is the effective coordination number, subscripts *i*, *j*, *k* denote Si (or C) atoms, $r_{ij}$ is the interatomic distance, $\theta_{ijk}$ is the angle between bonds i−j and j−k, and $g\left(\theta_{ijk}\right)$ is a function of this angle, which stabilizes the structure with covalent bonds. The parameters of the Tersoff potential for C–C and Si–Si interactions were taken from [59].

The modified potential of an embedded atom with the parameters reported in [60] were used to describe the interactions in nickel. The total energy defined by this potential is given as

(3) $$E_{tot}=\frac{1}{2}\sum_{i,j}V(r_{ij})+\sum_{i}F({\bar{\rho }}_{i}),$$

where $V\left(r_{ij}\right)$ is the pair potential depending on the distance $r_{ij}$ between atoms *i* and *j*; *F* is the embedding energy, which is a function of the host density; and $\bar{\rho_{i}}$ is induced at a site *i* by all other atoms in the system. The host density $\bar{\rho_{i}}$ is defined as

(4) $${\bar{\rho }}_{i}=\sum_{i\neq j}\rho (r_{ij}),$$

where $\rho \left(r_{ij}\right)$ is a function characterized as “atomic density”.

All other interactions were described by the Morse potential:

(5) $$\Phi (r)=D_{e}\left[\exp\{-2\alpha (r-r_{e})\}-2\exp\{-\alpha (r-r_{e})\}\right],$$

where $D_{e}$ is the depth of the potential well, α is the stiffness parameter, and $r_{e}$ is the equilibrium bond length. The potential parameters were reported in works [36,37,38,39,40,41] and were also obtained using simple interpolation relations [61].

The general expression for the binding energies in the silicene sheet and the nickel substrate, the adhesion energy between the silicene sheet and the nickel substrate, and the adsorption energy of lithium ad-atoms has the form:

(6) $$E=-\frac{E_{\mathrm{Tot}}-E_{1}-E_{2}}{N},$$

where *E*_Tot_ is the total energy of the entire system. Parameters *E*_1_, *E*_2_ and *N* take values depending on the calculated value. So, when calculating the adhesion energy: *E*_1_ and *E*_2_ are the energies calculated for a nickel or graphite substrate and a silicene sheet, respectively.

The roughness *R_a_* of the channel walls was determined through the average deviation of the coordinate z of the Si atoms in the sheet relative to the average (over the atoms forming the sheet) value of this coordinate,

(7) $$R_{a}=\frac{1}{N}\sum_{i=1}^{N}\left|z_{i}-\overset{-}{z}\right|,$$

Here, *N* is the number of nodes (atoms) on the silicene surface, $z_{i}$ is the z coordinate of atom i, and $\bar{z}$ is the average value of the z coordinate for the silicene structure; values of $z_{i}$ and $\bar{z}$ are determined at the same moment of time.

Calculation of stress distribution in silicene sheets is based on the division of sheets into elementary regions (stripes) with normal γ(x, y, z). These elementary stripes are elongated either in the armchair direction or in the zig-zag direction. A force acts on each of the stripes, the determination of which takes into account only those interactions between particles i and j, the force vector of which permeates the given stripe [34]. The directions α (x, y, z) for the velocities of atoms i and j are also taken into account:

(8) $$\sigma_{\gamma \alpha }^{}(l)=\langle \sum_{i}^{n}\frac{1}{\Omega }\left(mv_{\gamma }^{i}v_{\alpha }^{i}\right)\rangle +\frac{1}{S_{l}}\langle \sum_{i}^{n}\sum_{j\neq i}^{\left(u_{i}\leq u,u_{j}\geq u\right)}\left(f_{ij}^{\alpha }\right)\rangle .$$

Here, *n* is the number of atoms on the *l*th stripe, Ω is the volume per atom, *m* is the atomic mass, $v_{\alpha }^{i}$ is the α projection of the velocity of the *i*th atom, *S_l_* is the area of the *l*th surface element, $f_{ij}^{\alpha }$ is the *α* projection of the force resulting from the interaction of *i* and *j* atoms and passes though the *l*th stripe, and *u_i_* is the coordinate of the atom *i*; the symbol *u* denotes the coordinate of the contact point of the straight line through the centers of the atoms *i* and *j* and the *l*th surface element.

## 3. Results

### 3.1. DFT Simulation of Free-Standing and Doped Silicene

#### 3.1.1. Adsorption of Lithium on a Free-Standing Silicene Sheet

**Figure 1:**
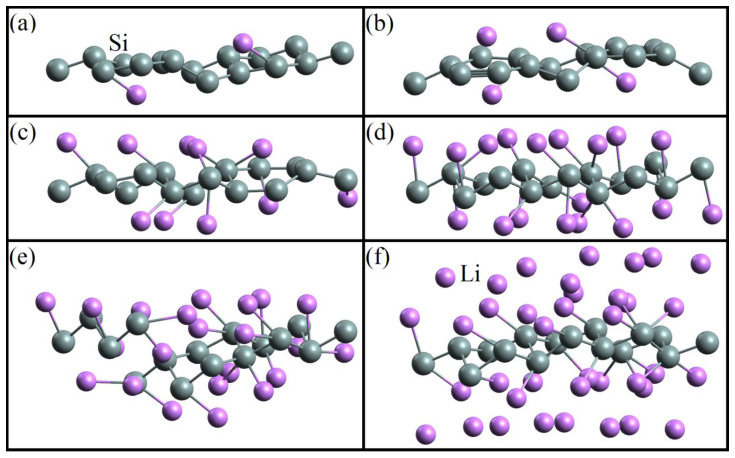
Geometric structure of the silicene/lithium system corresponding to the adsorption ratios: (**a**) 0.125, (**b**) 0.25, (**c**) 0.625, (**d**) 1, (**e**) 1.375, and (**f**) 2.375 [50].

In [50], sequential filling of the silicene sheet with lithium was performed. System configurations related to varying degrees of this filling are shown in Figure 1. Changing the number of Li atoms on the silicene resulted in the alteration of the Si-Si bond length. When the ratio *N*_Li_/*N*_Si_ reached 1.375, the incorporation of lithium atoms into the silicene sheet was observed (Figure 1e). This led to the formation of defects in silicene.

The change in the energy characteristics of the system associated with the number of Li atoms deposited on the silicene sheet is presented in Table 1. The bond between Si atoms becomes weaker (*E*^Si^_b_ = −4.279 eV) when the ratio *N*_Li_/*N*_Si_ = 1.375. This is due to the appearance of defects in the silicene sheet [47]. As lithium is filled, the character of the conductivity of silicene changes. In the majority of cases presented in Table 1, silicene acquires metallic conductivity (M). However, at *N*_Li_/*N*_Si_ = 0.25 and in the range 0.375 ≤ *N*_Li_/*N*_Si_ ≤ 1, the conductive properties of silicene disappear and it becomes a narrow-gap semiconductor.

#### 3.1.2. Free Doped Single and Double Layer Silicene

**Figure 2:**
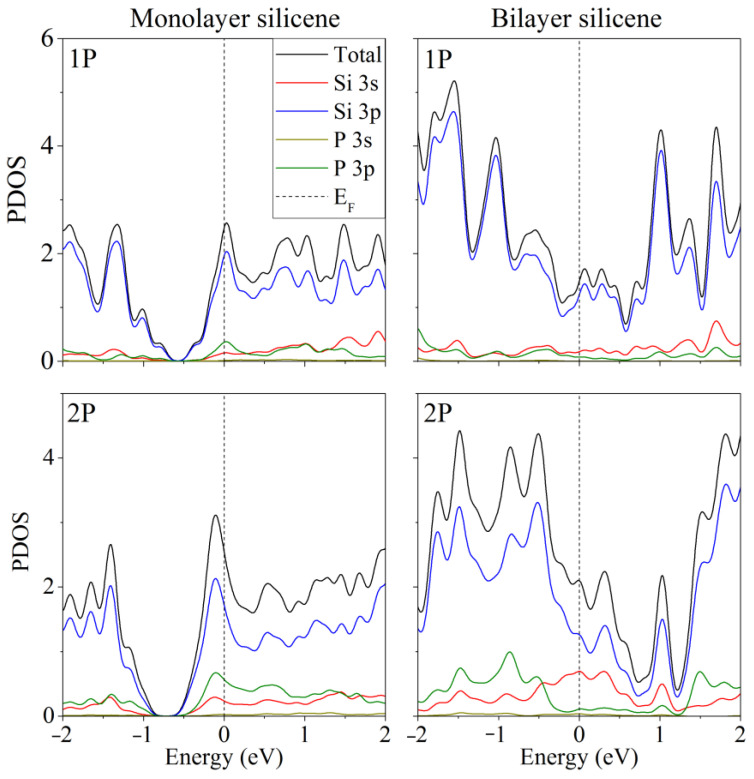
Partial spectra of electronic states of one- and two-layer silicene obtained by the replacement of 1 or 2 silicon atoms by phosphorus. Reprinted with permission from Ref. [48]. 2020, Springer.

**Figure 3:**
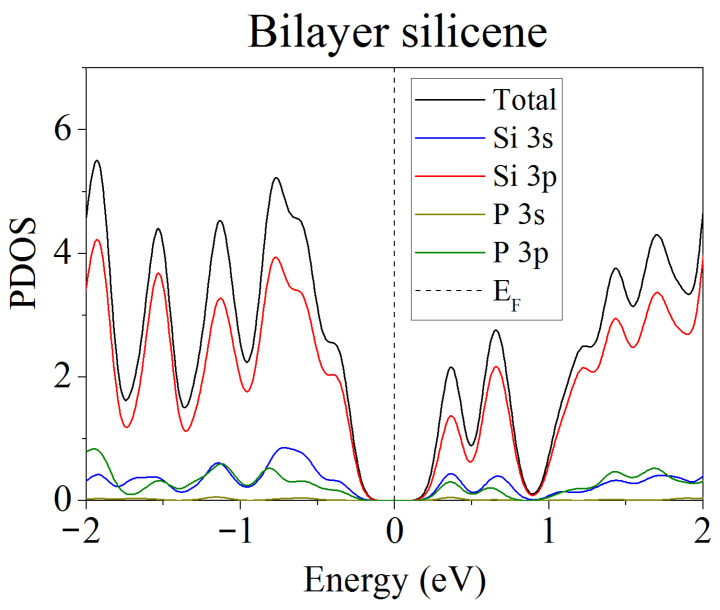
Partial spectrum of electronic states of two-layer silicene obtained after the replacement of 2 Si atoms belonging to each of the lower sublattices by phosphorus. Adapted with permission from Ref. [48]. 2020, Springer.

Doping of one- and two-layer free-standing silicene with phosphorus was studied in [48]. Representing them, the initial 2 × 2 supercells contained 8 and 16 Si atoms, respectively. In the quantum mechanical model, one or two Si atoms were replaced by P atoms. Electronic state spectra (PDOS) were calculated, the shape of which was used to track the change in the electronic properties of the doped two-dimensional material. In particular, it was noted that in most cases, as a result of doping, the energy gap between the valence band and the conduction band disappears, and silicene acquires conducting properties due to pp hybridization (Figure 2). However, in the case of two-layer silicene, the conductivity could not manifest itself when the substitution of Si atoms was carried out in the lower sublattices of the upper and lower layers of silicene. This procedure resulted in an increase in the band gap to 0.236 eV (see Figure 3).

#### 3.1.3. Doped One- and Two-Layer Silicene on a Graphite Substrate

**Figure 4:**
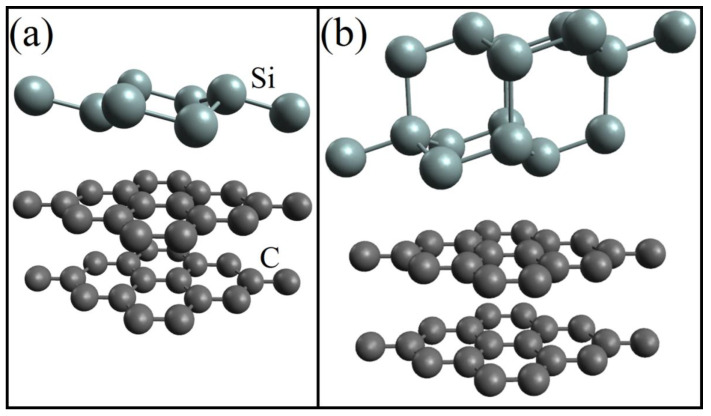
Geometric structure of (**a**) one- and (**b**) two-layer silicene on a graphite substrate after geometric optimization. Reprinted with permission from Ref. [46]. 2020, Springer.

A DFT study of the electronic properties of doped silicene on a graphite substrate was performed in [46]. The number of C atoms in the two-layer graphite substrate was 36. Undoped systems with one- and two-layer silicene were preliminarily studied. The corresponding configurations obtained after geometric optimization are shown in Figure 4. After optimization, the distances between sublattices in each of the layers of two-layer silicene increased significantly, while the similar distance in single-layer silicene even slightly decreased.

After silicene doping with phosphorus and carbon substrate with nitrogen, an increase in the binding energy was observed in both modified subsystems. In the Si(P) subsystem, the binding energy increased by 2–15%, and in the C(N) subsystem, by ~2%.

**Figure 5:**
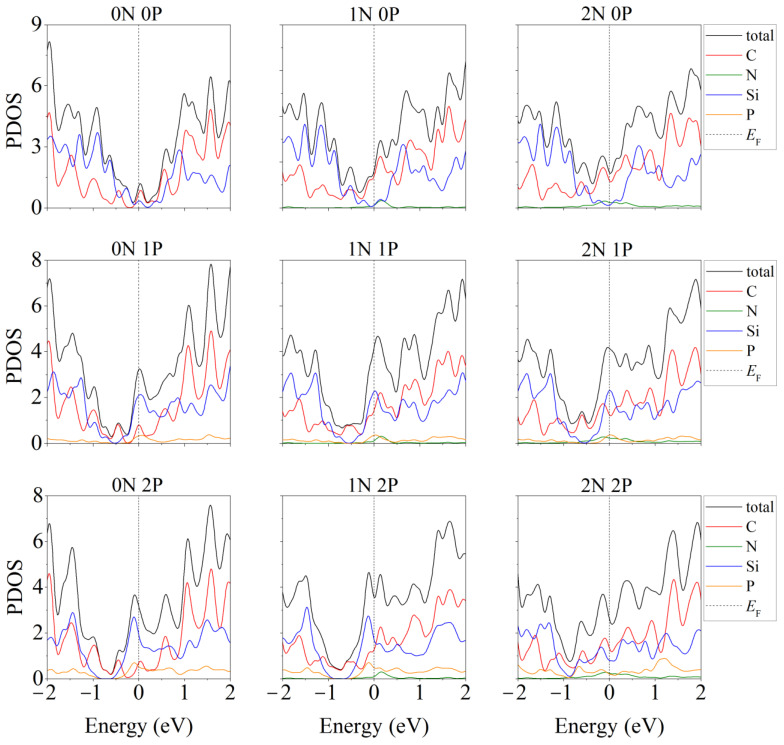
Partial densities of electronic states of doped systems “two-layer silicene/graphite substrate” at various degrees of doping with P and N. Reprinted with permission from Ref. [46]. 2020, Springer.

The partial densities of electronic states for this system are shown in Figure 5 [46]. As a result of nitrogen substrate doping, electronic conductivity always appeared in the system, regardless of whether silicene is doped. The same effect was achieved in the presence of one P atom in silicene. In this case, the substrate could be undoped. However, among the “two-layer silicene/carbon substrate” systems containing two phosphorus atoms, there may be semiconductors.

**Figure 6:**
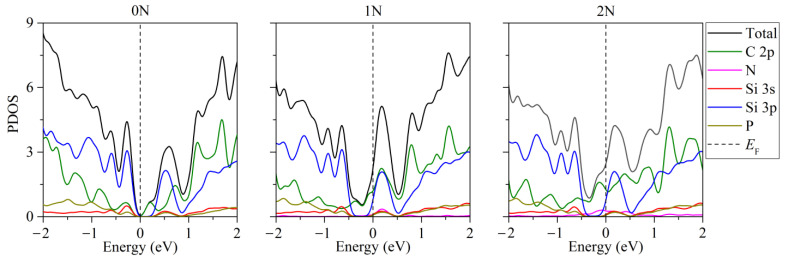
Partial densities of electronic states of doped systems “two-layer silicene/graphite substrate” in the presence of 2 P atoms in silicene. Reprinted with permission from Ref. [46]. 2020, Springer.

In the presence of two phosphorus atoms in the “two-layer silicene on a carbon substrate” system, when there are zero to two nitrogen atoms in the substrate, the partial densities of electronic states have the form illustrated in Figure 6 [46]. Extremely low electronic conductivity is observed in the absence of substrate doping. In this case, the band gap can range from 0.008 to 0.018 eV (Figure 6, 0 N). Whether the system will have the properties of a semiconductor depends largely on the arrangement of the P atoms in the silicene. In particular, this is realized in the case of an undoped carbon substrate for the following P localizations: 1. two atoms in the lower sublattice of the lower silicene sheet; 2. two atoms in the upper sublattice of the lower silicene layer; 3. one atom in the lower and one atom in the upper sublattice of the lower sheet of silicene; 4. one atom in the lower sublattice of the lower sheet and one atom in the upper layer of silicene. The PDOSs for cases 3 and 4 are almost identical. The phosphorus-doped silicene system always became conductive in the presence of nitrogen in the carbon substrate (Figure 6, 1 N, 2 N).

#### 3.1.4. Transmutation Doping of Silicene on a Nickel Substrate

The influence of the thickness of the FCC nickel substrate with the (001) face towards silicene on the adhesion of silicene to Ni was studied in [49]. The difference in adhesion energies in the presence of four and five layers of atoms in the substrate was ~1%. Therefore, the main calculations were performed for a four-layer substrate containing 36 Ni atoms.

**Figure 7:**
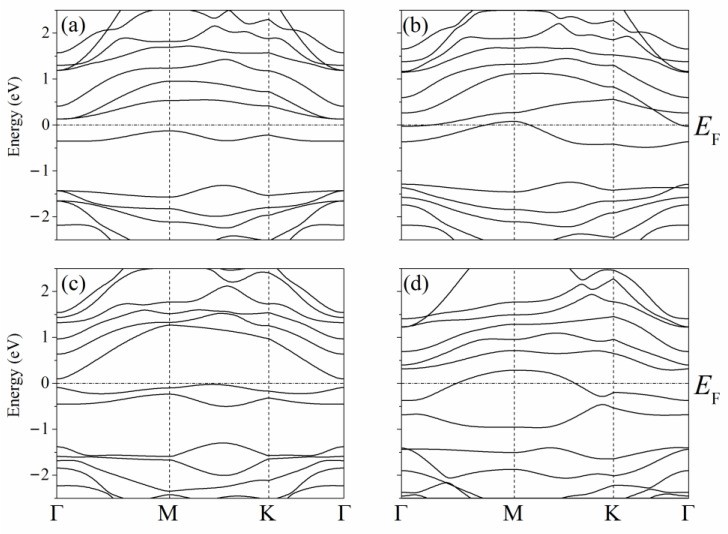
Band structures of silicene modified with one (**a**,**b**) or two (**c**,**d**) phosphorus atoms upon adsorption of one lithium atom in a position above the center of the six-membered ring; (**a**) and (**b**)—P atom is in the lower or the upper sublattice of silicene, respectively, (**c**)—two P atoms are in the lower sublattice or two P atoms are in the upper sublattice, (**d**)—one P atom is in the upper sublattice, and the other P atom is in the bottom one. Reprinted with permission from Ref. [45]. 2021, Elsevier.

The band structure of the system described above with one adsorbed Li atom on single-layer silicene has the form shown in Figure 7 [45]. Due to the presence of P atoms in silicene, a semiconductor–conductor transition can be observed in the system under consideration. As in the case of a carbon substrate, the conductivity of silicene on a nickel substrate is largely determined by the arrangement of P atoms in silicene. In the presence of one P atom in silicene, the resulting indirect band gap has a value of Δ = 0.256 eV (Figure 7a,b). The band gap decreases when two Si atoms in silicene are replaced by P atoms when both P atoms are located either in the lower or upper sublattice Δ = 0.023 eV. However, when one of them is in the lower sublattice and the other in the upper sublattice, Δ = 0.122 eV (Figure 7c,d, respectively).

**Figure 8:**
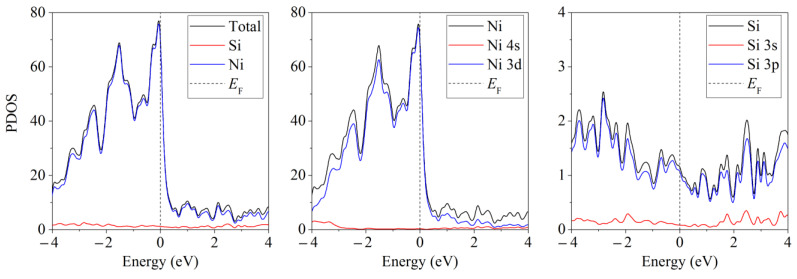
Partial spectra of electronic states of the “silicene/nickel substrate” system; the substrate contains 4 layers of Ni. Reprinted with permission from Ref. [45]. 2021, Elsevier.

Unlike free-standing silicene, which is a narrow-gap semiconductor (Δ = 0.027 eV) [62], silicene on a nickel substrate in the absence of doping has barrier-free electronic conductivity. Detailed partial spectra of the electronic state density of such a system are presented in Figure 8 [45]. The conductivity in this case is explained by the interaction of Ni 3d electrons with Si 3p electrons.

Thus, ab initio calculations show that silicene on a graphite substrate after neutron transmutation doping transforms from a narrow-gap semiconductor into a conductive material; however, if silicene is two-layered, then semiconductor properties can be pre-served. The adhesion energy between silicene and a graphite substrate is significantly inferior to that between silicene and metal substrates, which contributes to the experimental production of a “silicene/graphite” heterostructure. Calculation of the electronic structure of the “silicene/nickel substrate” system shows that after the NTD procedure, the conductivity of this system is preserved. The adhesion energy between silicene and nickel substrate is the highest with respect to *E*_adh_ with other metal substrates (Ag, Al, Cu) and graphite. This is one of the limiting factors in the experimental preparation of silicene on a nickel substrate.

### 3.2. Classic MD Modeling of Doped Silicene Anode

#### 3.2.1. The Specific Energy of the Lithium-Ion Battery with Combined Silicon Anode

**Figure 9:**
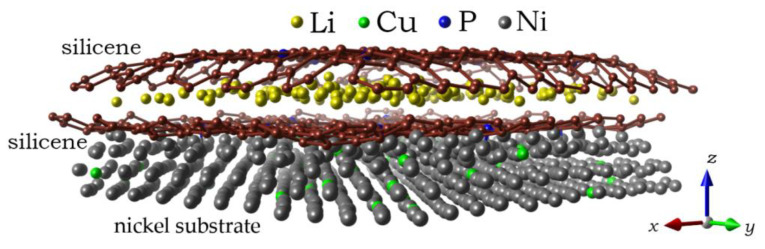
A lithium-filled channel with a 0.75 nm gap between the silicene sheets after 150 ps; the channel walls are doped with 3% phosphorus; a Ni substrate with 5% doping with Cu is used; the figure shows only the three closest to silicene channel layers of the metal substrate. Reprinted with permission from Ref. [39]. 2021, Elsevier.

Classical MD modeling allows us to consider larger systems. In particular, this makes it possible to observe changes in the shape of silicene sheets, between which Li atoms are embedded. Initially, parallel silicene sheets formed a flat channel, the walls of which were doped with phosphorus in the following percentages of the number of P atoms with respect to Si atoms: 3%, 6%, 9%, and 18%. In fact, P atoms were inserted into preliminarily created mono-, bi-, tri-, and hexavacancies uniformly distributed over the silicene sheets. The nickel substrate was doped with Cu atoms by random selection of Ni atoms to be replaced. The channels constructed in this way were filled with Li^+^ ions displaced by an electric field. After certain time intervals, the ions that entered into the channel acquired electrons and transformed into Li atoms. Figure 9 shows a lithium-filled, wall-doped (3% P) silicene channel, which is located on a Ni substrate with 5% Cu doping [39]. Three bottom layers of the substrate, which do not influence the channel filling process, are not shown in the figure. As the channel is filled with lithium, it acquires a convex shape.

**Figure 10:**
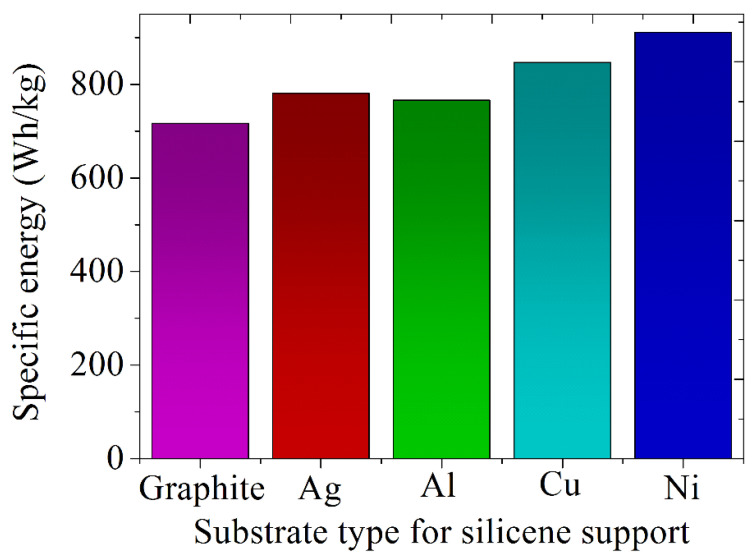
Estimated specific energies of batteries with a silicene anode placed on various types of substrates -. Reprinted with permission from Ref. [63]. 2022, Ural Federal University&Institute of High Temperature Electrochemistry, UB RAS.

The specific energies of batteries with silicene anodes on metal (Ag, Al, Cu, Ni) and graphite substrates in the absence of doping were determined using the classical MD modeling method [63]. These data are presented in Figure 10, which shows that the substrate has a significant impact on this important battery characteristic. In particular, the use of a Ni substrate can increase the specific energy by 27% compared to the case of using a graphite substrate. The use of silicene anodes can increase the specific energy of the best modern batteries by 4–5 times on average.

#### 3.2.2. Lithium Intercalation Effects When a Silicene Channel Is on a Ni(111) Substrate

**Figure 11:**
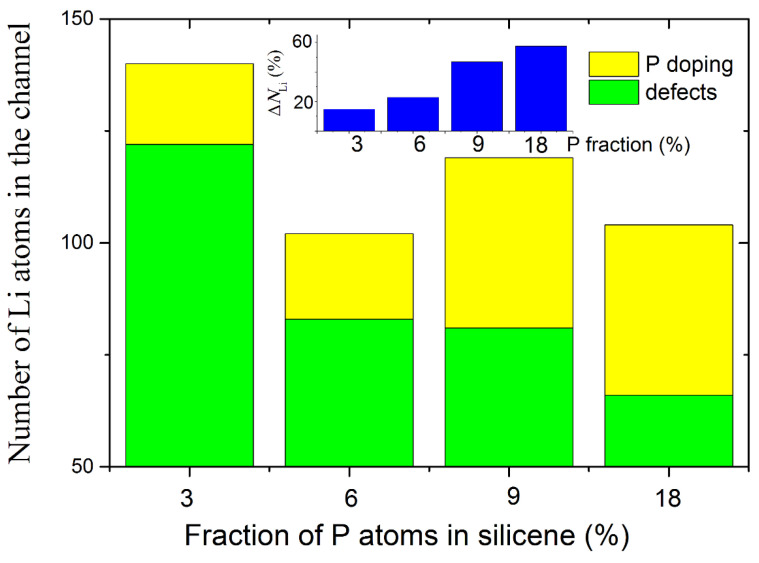
Total number of Li atoms successfully incorporated into the silicene channel in the case of the doped systems; an inset illustrates a change in channel capacitance after doping; the channel is located on a nickel substrate doped with 5% Cu. Adopted with permission from Ref. [39]. 2021, Elsevier.

The effect produced by doping the walls of the silicene channel with phosphorus in the presence of a copper-doped nickel substrate, on the degree of its filling with lithium, is shown in Figure 11 [39]. As can be seen from the figure, the increase in the filling of the channel with lithium can be from 15% to 57%, depending on the degree of doping of the channel walls. An increase in the degree of doping leads to an increase in the doping-initiated contribution to channel occupancy. However, high doping reduces the mechanical stability of the channel walls, so that at 9% and 18% doping, the channel tends to break. Therefore, 3% doping of the silicene channel walls with phosphorus turns out to be optimal.

**Figure 12:**
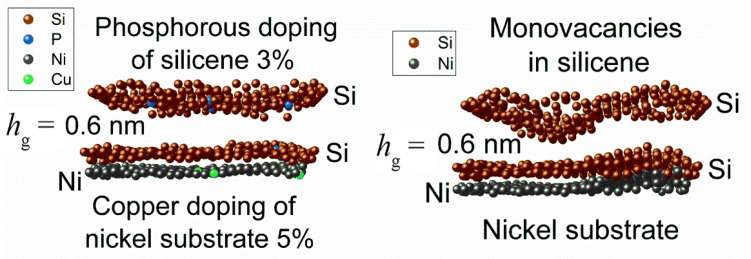
Zx-projections of the silicene channel (doped with phosphorous or modified with vacancies) and the top sheet of the Ni(111) substrate. Adopted with permission from Ref. [39]. 2021, Elsevier.

Figure 12 shows the zx-projections of the 0.6 nm gap silicene channel, together with the top layer of the substrate [39]. The system on the left was doped, while the system on the right was not. The walls of the doped silicene channel contain 3% P atoms, while the undoped silicene walls contain 3% monovacancies. As can be seen from the figure, the doped silicene channel is deformed during lithium intercalation much weaker than the undoped channel. In both cases, the bottom sheet of silicene is less curved than the top sheet. Therefore, the adhesion between the bottom sheet of silicene and the substrate is sufficient to have a stabilizing effect on the change in the shape of this sheet.

**Figure 13:**
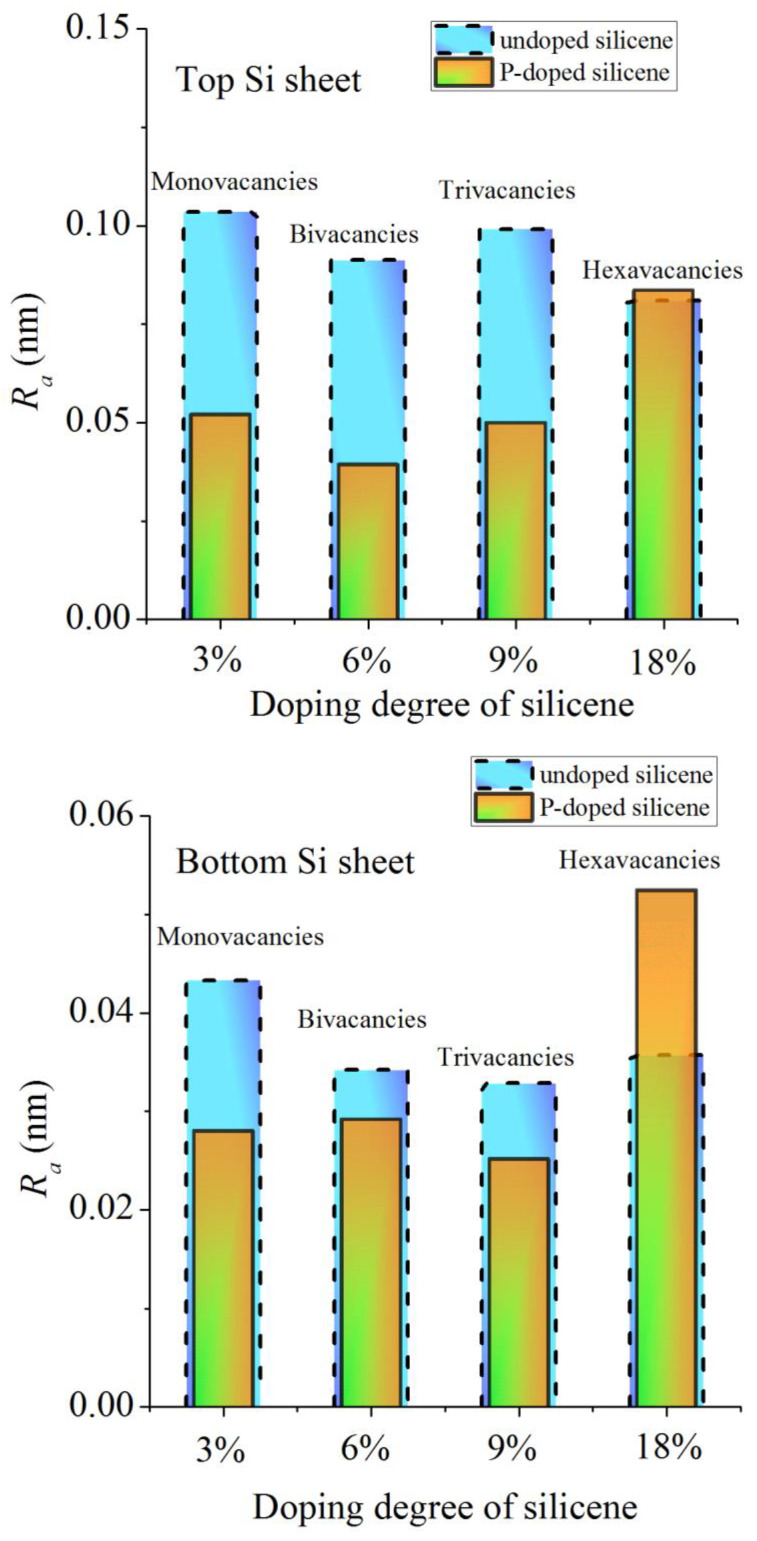
Calculated values of the roughness of the sheets of the silicene channel depending on the degree of doping of its walls with phosphorus (orange bars) or on the size of vacancies (blue bars); the Ni(111) substrates are 5% Cu doped and undoped in the presence of vacancies in silicene, respectively. Adopted with permission from Ref. [39]. 2021, Elsevier.

Figure 13 compares the calculated roughness for channel walls doped with phosphorus and undoped walls containing monovacancies [39]. In the first case, the Ni(111) substrate was 5% Cu doped, while in the second case, the substrate was not doped. In both cases, the bottom sheet of silicene appears less rough than the top sheet. With doping levels up to 9%, there is a significant reduction in roughness for doped silicene sheets, especially for the top sheet. However, when the doping rate reaches 18%, the doped silicene sheets become rougher than the corresponding undoped sheets. At this doping level, this is due to the displacement of P atoms into the channel. This displacement creates an obstacle to the movement of Li atoms along the channel, as a result of which the walls of the channel are significantly bent and tend to be destroyed.

#### 3.2.3. Lithium Intercalation and Physical Properties of Doped Two-Layer Silicene Which Is on a Graphite Substrate

**Figure 14:**
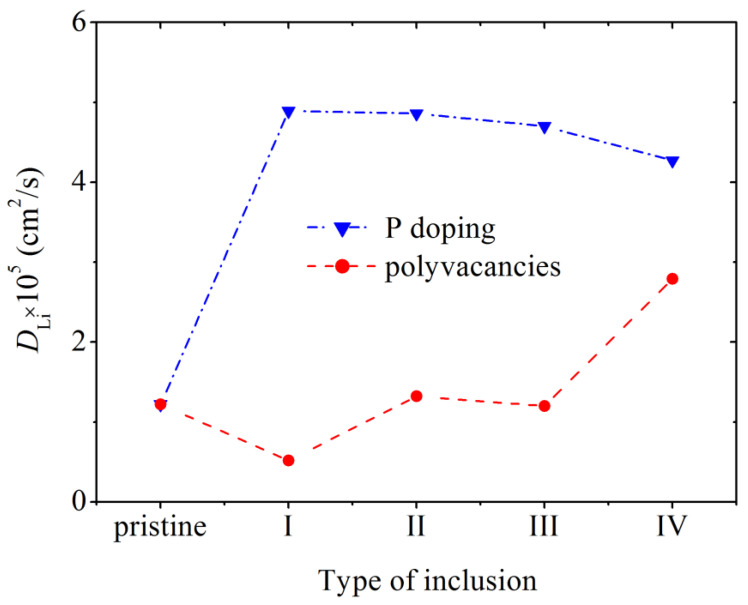
Diffusion coefficient of Li ions during their movement along a pristine silicene channel and a channel whose walls contain P atoms and polyvacancies: I, II, III, IV—mono-, bi-, tri-, and hexavacancies in silicene, respectively; in all cases, the silicene channel is located on a graphite substrate with a nitrogen content of 5%. Adopted with permission from Ref. [11]. 2021, Elsevier.

The mean square displacement $\langle \Delta r^{2}\rangle$ of Li atoms was calculated in a defect-free silicene channel, as well as in channels with P-doped walls and walls with polyvacancies, in the classical MD model. The values of coefficient *D*, defined through $\langle \Delta r^{2}\rangle$, in a doped channel are much higher than those in channels with walls containing polyvacancies and with pristine walls (Figure 14) [11]. Doping causes a smoothing of the silicene buckles, which enhances the self-diffusion of Li atoms in the channel. The maximum value of *D* is reached at 3% doping with phosphorus. The penetration of P atoms into the channel, the walls of which are more heavily doped, weakens the self-diffusion of Li atoms. The low value of *D* in channels with walls having polyvacancies is associated with a significant deformation of the walls during lithium intercalation. The coefficient *D* increases in the presence of hexavacancies in the channel walls, since Li atoms leave the channel through such holes.

**Figure 15:**
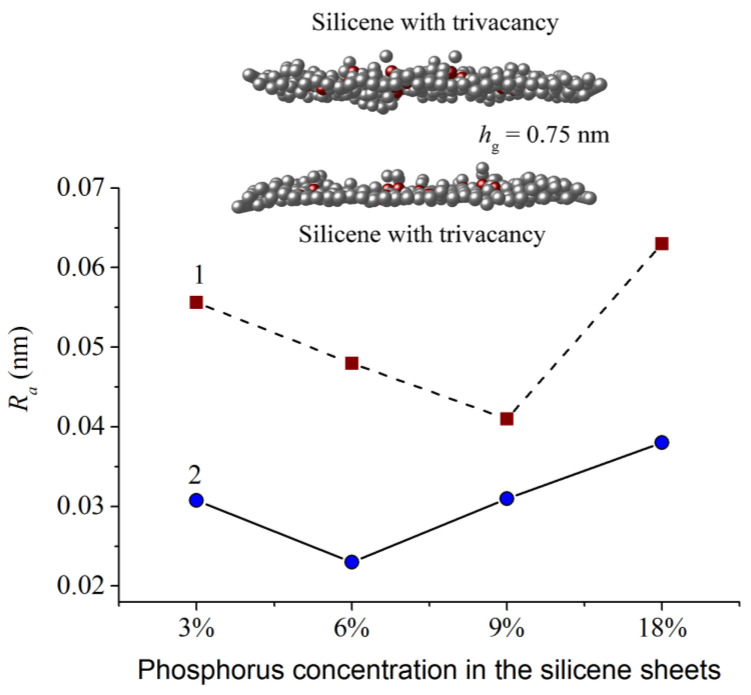
Roughness of the top (1) and bottom (2) silicene sheets of the system “P-doped silicene channel located on the 5% N-doped graphite substrate” with different phosphorus concentrations; insert shows *zx*-projection of the silicene channel with the 9% phosphorus concentration. Reprinted with permission from Ref. [11]. 2021, Elsevier.

The roughness *R_a_* of the phosphorus-doped walls of the silicene channel after lithium deintercalation, depending on the degree of doping of the silicone sheets, is shown in Figure 15 [11]. As can be seen from the figure, the channel wall adjacent to the substrate has a significantly lower roughness than the upper wall. The highest *R_a_* value is observed for silicene walls doped with 18% phosphorus. Such a channel tends to collapse. The upper part of the figure shows the zx-projection of the silicene channel doped with 9% phosphorus. At this level of doping, the value of *R_a_* for the upper wall of the channel is minimal.

**Figure 16:**
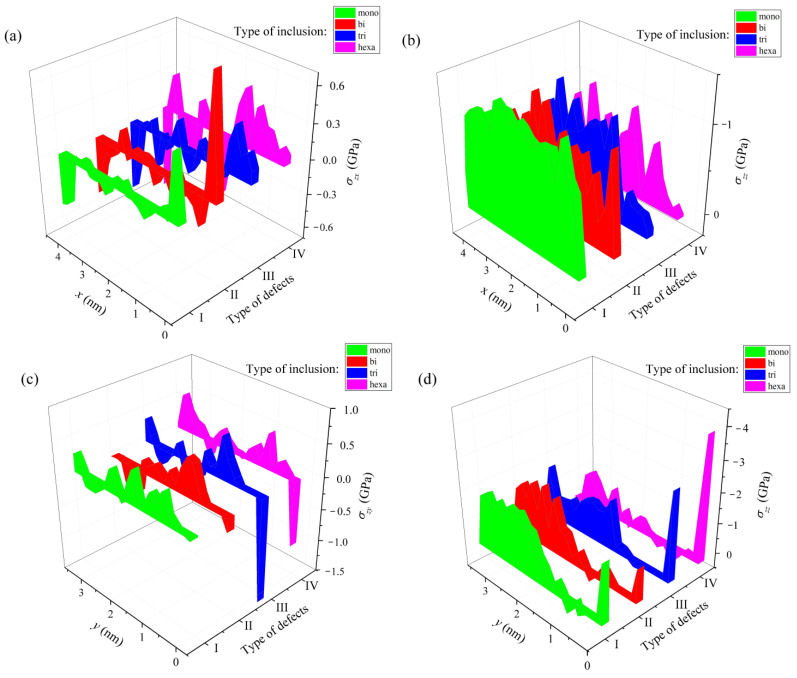
Stress distribution in a lithium-intercalated silicene channel with defects initially filled with P atoms; the channel with a gap of 0.24 nm is located on a graphite substrate, which has 5% doping with nitrogen; (**a**,**b**) show the distribution of stresses *σ_zx_* and *σ_zz_* along the direction 0 *x* (zig-zag); (**c**,**d**) show the distribution of stresses *σ_zy_* and *σ_zz_* along the direction 0 *y* (armchair); types of defects filled with P atoms: I, mono-, II, bi-, III, tri-, and IV—hexavacation. Reprinted with permission from Ref. [46]. 2020, Springer.

In [46], the average stress tensor describing the nature of the deformations in doped silicene sheets after lithium intercalation into a channel located on a graphite substrate was calculated. The channel gap in this case was 0.24 nm. As in the case of metal substrates, the most significant stress on the walls of the silicene channel is the stress σ_zz_. As a rule, the strongest stresses occur at the edges of the sheets. The most significant stresses σzz exist on one of the edges in the armchair (0 y) direction at 9% and 18% P doping (Figure 16d). However, even these local stresses are much lower than the value of tensile strength of silicene (23 GPa) [11]. The distribution of stresses measured in both directions (zig-zag and armchair) is highly non-uniform. A more uniform stress distribution is observed when moving in the zig-zag (0 x) direction with 3% doping of the channel walls.

Experimental and computational studies have shown that the thermal conductivity in silicon nanostructures and porous silicon can be reduced by two orders of magnitude compared to their solid bulk counterparts [64,65]. Various types of point defects, such as interstitial atoms, substitutional atoms, vacancies, and ion irradiation, effectively reduce the phonon thermal conductivity of nanomaterials [66]. The effect of doping and various vacancy defects on the thermal conductivity of silicene is difficult to demonstrate practically at the nanoscale. However, computer studies make it possible to reveal such changes in the properties of silicene. In [67], a non-equilibrium molecular dynamics simulation was performed to calculate the phonon thermal conductivity of silicene using the optimized Tersoff potential. The influence in various parameters, such as temperature, concentration of dopant-carbon, and vacancy defects, on the thermal conductivity of silicene has been studied. Carbon was chosen as an alloying element, as a representative of the same group IV as silicon. In addition, carbon is stable in the silicene matrix, and the difference in the masses of these elements makes the doping effect more noticeable. The predicted thermal conductivity value of pristine silicene at room temperature was ~20 W/m·K. A sharp decrease in the thermal conductivity of silicene with an increase in temperature from 100 K to 600 K was found. Removing only 1% of the silicon atoms from the original nanosheet leads to a more than 50% decrease in the thermal conductivity of both pure and carbon-doped silicene.

Thus, the gravimetric energy density or the specific energy of a battery with a “silicene/Ni” anode is superior to that of lithium-ion batteries with other combined anodes (silicene/{graphite, Ag, Al, Cu}). Doping the silicene anode with phosphorus increases the performance of the LIB without the risk of failure. At all doping levels considered, the self-diffusion coefficient of lithium atoms in the channel increases significantly compared to the case of a channel with undoped walls. This means that moderate doping (up to 6%) of the silicene anode should noticeably increase the charging rate of the battery. The graphite substrate subjected to the NTD procedure reduces the corrugation of the anode silicene sheet in direct contact with it and does not increase the corrugation of the silicene sheet following it. Despite weak adhesion, the graphite substrate does not adversely affect the mechanical stability of the 2D silicon material during LIB operation. The reduction in the height of bends in silicene due to alloying contributes to an increase in the filling of the modified channel located on graphite with lithium. At the same time, the stresses arising when the channel is filled with lithium are small compared to the tensile strength of silicene.

## 4. Discussion

This review presents computational and theoretical studies on silicene strengthening. The ways to improve silicene-containing materials for their use as a LIB anode material have been outlined. A NTD method is proposed to effectively solve the problem of increasing the capacity of LIBs without a significant decrease in the strength of the anode material. The conditions that would allow achieving the metallization of silicene on graphite and nickel substrates, which can increase the rate of battery charging, are considered. The deformations of two-layer silicene, which appear as a result of lithium intercalation, are studied. The stabilizing role of the substrate in this process has been established, due to which the height of the buckles on the silicene is reduced. The influence of the substrate material and the type of vacancy defects on the filling of a silicene channel with lithium and on its mechanical stability is studied. The dependence of the self-diffusion coefficient of lithium atoms on the level of P doping and the type of vacancy defects in the walls of the silicene channel has been established. However, these studies are not yet sufficient to successfully predict the suitability of the modified two-dimensional material for constructing the LIB anode. Thermal conductivity shall be included as the tested property of the two-dimensional material. The existing experimental and calculated data indicate that the thermal conductivity of silicene is significantly reduced when it is modified [64,65]. Low thermal conductivity can become an obstacle to the use of silicene on any of the substrates as an anode material.

In general, there is a whole range of interrelated problems in the creation of a new generation of LIBs that should be resolved in the near future. We will consider only those of them for which the solution, in our opinion, is of the greatest importance.

A sharp irreversible loss of capacitance and low Coulomb efficiency, which is due to the mechanical destruction of massive silicon anodes, are the key problems for their commercial use. In addition, delithiation destroys the solid electrolyte interface (SEI). As a result, the fresh silicon surface interacts with the electrolyte, forming a new SEI site, which increases the thickness of the SEI with each charge/discharge cycle, resulting in a decrease in LIB efficiency.

The doping of anode materials for LIBs performed to date generally results in high specific capacitance and high energy density. In this case, however, the cyclic characteristics of LIBs do not improve [68]. More stable cyclic performance is shown by LIBs with anodes having some special morphology (e.g., nanospheres and nanowires) or when the negative electrode is modified with graphene or carbon foam.

Diffusion of salts limits the performance of electrochemical cells with thick electrodes. To date, solid electrolytes based on sulfides have been found to have a higher ionic conductivity than their liquid counterparts. However, sulfide electrolytes, as a rule, have insufficient electrochemical stability, toxicity, and flammability problems [69]. In addition, the energy density of all-solid-state batteries is still low.

Currently, the manufacture of silicene is associated with significant problems. In particular, it requires an ultra-high vacuum environment, while suitable substrates are still being selected. Therefore, it is important to gain a deeper understanding of the physical and chemical properties of silicene.

We consider it important to solve the following problems for further effective modification of the anode material. To successfully carry out nanoscale design, it is necessary: first, to investigate the dependence of the properties of a nanostructure on the size of its elementary units; second, to establish the real reasons for the decrease in the Coulomb efficiency during the first cycle, including the formation of the SEI film [70]; third, to study more carefully the microstructural changes that occur during lithiation/delithiation (to understand the electrochemical processes inherent in these changes, it is necessary to use DFT and classical MD modeling); and fourth, the choice of an appropriate electrode design, as well as the choice of electrolyte, is no less important.

In addition, in the struggle to increase the capacity of the electrode, the synergistic effect of the cathode and anode, which can maximize battery performance, should not be discounted. Under no circumstances should the structural stability and safety of the battery be ignored. At the same time, toxic and harmful substances cannot be used as electrode materials. It must be ensured that the materials used in the LIB can be easily disposed after the end of operation to prevent any harm to the environment.

## 5. Conclusions

Thin doped films of silicon are the most promising anode material for next-generation lithium-ion batteries. Their use in anode design can accommodate the volume expansion during cycling, thus leading to stable battery cycling. In addition, such anodes should provide high theoretical specific capacity and safe electrochemical potential. Computational methods are becoming increasingly important in the search for high-energy-density and high-capacity anode materials. These data are needed to optimize the performance of LIBs. We hope that our research and subsequent work, where similar methods will be applied, will provide an understanding of non-equilibrium phenomena in thin-film electrodes and will guide the design and synthesis of electrode materials for high-performance lithium-ion batteries.

## Figures and Tables

**Table 1:** Dependence of the energy characteristics * of a free-standing silicene sheet adsorbed by lithium on the number of deposited Li atoms.

*N*_Li_/*N*_Si_	*E*_b_, eV	*E*^Si^_b_, eV	*E*^Li^_b_, eV	*E*_adh_, eV	BG, eV
0.0625	−4.631	−4.786	-	2.154	M
0.125	−4.491	−4.745	−0.236	2.219	M
0.1875	−4.375	−4.685	−0.252	2.474	M
0.25	−4.273	−4.658	−0.511	2.223	0.411
0.3125	−4.161	−4.677	−0.548	1.961	M
0.375	−4.075	−4.649	−0.686	1.858	0.474
0.625	−3.782	−4.596	−0.908	1.574	0.134
0.875	−3.599	−4.557	−1.088	1.417	0.409
1	−3.541	−4.520	−1.172	1.390	0.645
1.125	−3.408	−4.551	−1.180	1.212	M
1.375	−3.250	−4.279	−1.275	1.227	M
1.625	−3.052	−4.518	−1.278	0.871	M
1.875	−2.942	−4.492	−1.343	0.772	M
2.125	−2.832	−4.520	−1.385	0.652	M
2.375	−2.755	−4.530	−1.407	0.601	M

* *E*_b_ is the total binding energy of all atoms in the system; *E*^Si^_b_ is the binding energy of silicon atoms in a silicene sheet; *E*^Li^_b_ is the binding energy between lithium atoms in the lithium subsystem; *E*_adh_ is the adhesion energy between lithium and silicene sheet; BG is the band gap.

## Data Availability

Not applicable.
